# Case Report: Alpha-fetoprotein-producing urothelial carcinoma of the bladder

**DOI:** 10.3389/fonc.2025.1581665

**Published:** 2025-05-08

**Authors:** Haibing Huang, Youping Ding, Yuju Fang, Kunlin Xie, Chengyong Zhong, Haiye Fan, Xiaofeng Zou, Tianpeng Xie

**Affiliations:** ^1^ First Clinical Medical College, Gannan Medical University, Ganzhou, China; ^2^ Center of Medical Big Data and Bioinformatics Research, First Affiliated Hospital of Gannan Medical University, Ganzhou, China; ^3^ Department of Urology, First Affiliated Hospital of Gannan Medical University, Ganzhou, China; ^4^ Department of Pathology, First Affiliated Hospital of Gannan Medical University, Ganzhou, China

**Keywords:** alpha-fetoprotein, urothelial carcinoma, bladder cancer, radical cystectomy, case report

## Abstract

Elevated serum levels of alpha-fetoprotein (AFP) are commonly associated with hepatocellular carcinoma or germ cell tumors. AFP-producing urothelial carcinoma of the bladder is very rare. We report a case of bladder urothelial carcinoma with significantly elevated AFP levels. A 60-year-old man was admitted for gross hematuria. Computed tomography urography revealed multiple large tumors of the bladder. The serum level of AFP was up to 2,329 ng/ml. No hepatic tumors or testicular tumors were detected. Although the hepatitis B surface antigen test was positive, the liver function test was normal. The patient underwent robotic-assisted laparoscopic radical cystectomy with ileal conduit urinary diversion. Postoperative pathology revealed high-grade muscle invasive bladder urothelial carcinoma and immunohistochemical staining of the tumor cells showed strong AFP positivity. After surgery, the patient’s serum AFP levels dropped sharply and decreased to normal 4 weeks after the operation. In conclusion, AFP-producing bladder urothelial carcinoma is rare, and the mechanism and pathophysiology remain unclear and require further investigation.

## Introduction

Elevated serum levels of alpha-fetoprotein (AFP) are typically associated with hepatocellular carcinoma (HCC) or germ cell tumors. Other AFP-producing tumors including gastric cancer, ovary cancer, and lung cancer have been reported (1–3). Bladder urothelial carcinoma with AFP elevation is rare and has rarely been reported. Here, we report a case of bladder urothelial carcinoma with significantly elevated AFP levels and review the literature to discuss the diagnosis, treatment, and prognosis.

## Case presentation

**Figure 1:**
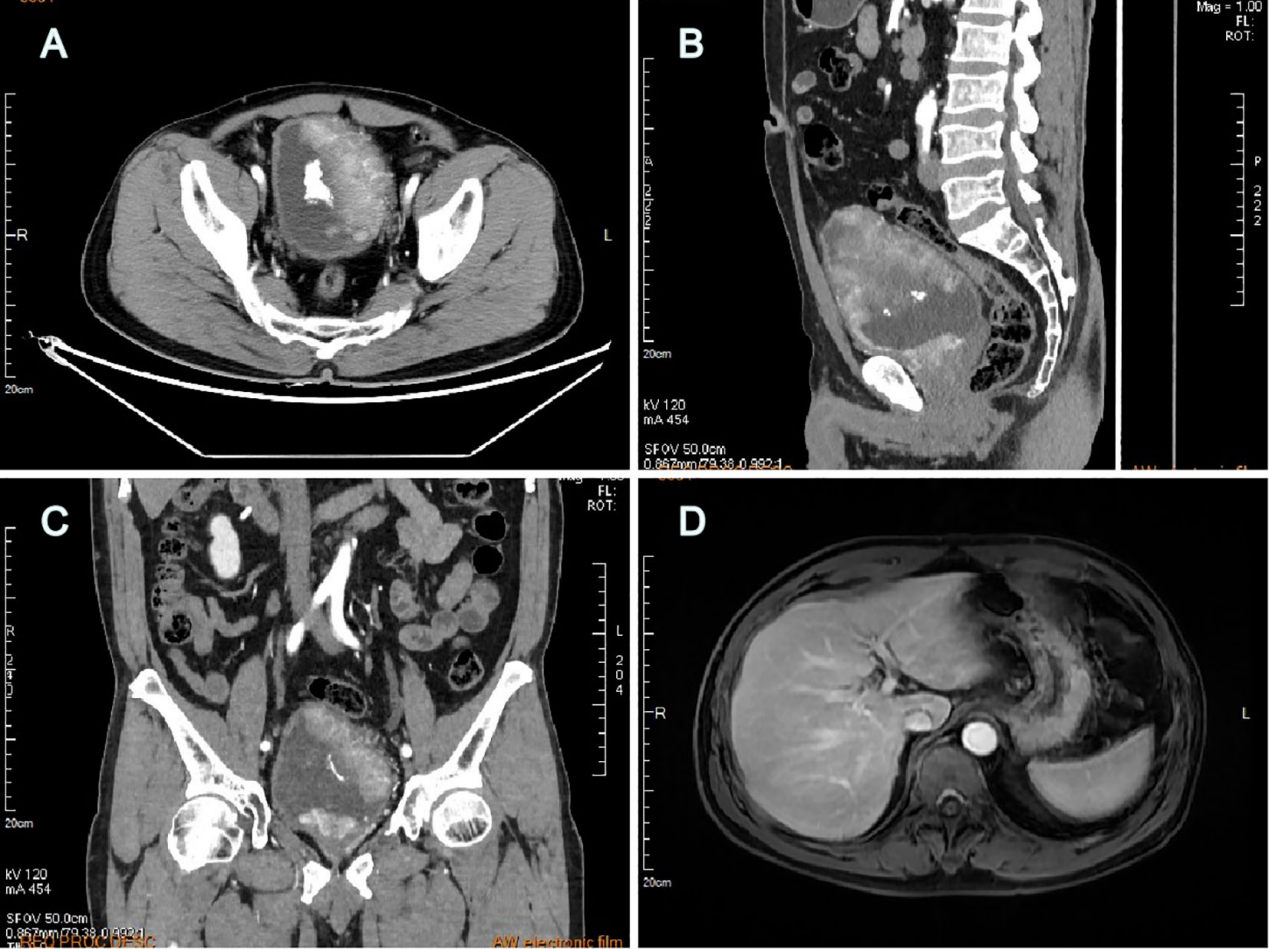
Computed tomography urography revealed multiple large lesions of the bladder **(A)** transverse plane, **(B)** sagittal plane, **(C)** coronal plane; Abdominal MRI with contrast revealed no detectable liver lesions **(D)**.

A 60-year-old man with intermittent gross hematuria and frequent urination for over 2 months was found to have multiple bladder-occupying lesions on ultrasonography at the local hospital and then presented to our hospital. Physical examination revealed a large hard immobile mass in the middle of the lower abdomen while his liver, gallbladder, spleen, kidneys, prostate, testis, and epididymis were unremarkable. Urinalysis showed a red blood cell (RBC) count of 995.35/ul, white blood cell (WBC) count of 26.05/ul, and protein of +1. Computed tomography urography (CTU) revealed irregular thickening of the bladder wall with multiple lesions, the largest measuring 97mm x 48mm, with unclear boundaries and calcifications on the surface. The lesions showed significant enhancement on contrast imaging, with extension into the prostatic urethra (**Figures 1A–C**). Hepatitis B surface antigen (HBsAg), hepatitis B e-antibody (HBeAb), and hepatitis B core antibody (HBcAb) were positive. Hepatitis B surface antibody (HBsAb), hepatitis B e-antigen (HBeAg), and hepatitis C virus antibody (HCV-Ab) were negative. The hepatitis B virus DNA (HBV-DNA) quantitative result was 1.75x10^5^ IU/ml. Liver, renal, and coagulation function tests were all normal. Serum AFP was 2,329ng/ml and remained significantly elevated at 1,802ng/ml after anti-HBV treatment with tenofovir pofol fumarate for 1 week before surgery. PSA, CEA, CA125, CA153, and CA199 were all within normal limits. The AFP isoform L3 percentage was 34.8%, and the GALAD score, which determines risk of HCC based on patient sex, age, and serum levels of AFP, AFP-L3, and des-gamma-carboxy prothrombin (DCP), was ≥2.57. Ultrasound of the digestive system showed a fatty liver and a 24mm x 15mm hypoechoic area in the left lobe of the liver, suggesting uneven fat distribution. Liver transient elastography showed a Controlled Attenuation Parameter (CAP) value of 233dB/m and a stiffness E value of 4.3kPa, both within normal limits. Abdominal magnetic resonance imaging (MRI) with contrast revealed no significant abnormalities in the liver, gallbladder, pancreas, or spleen (**Figure 1D**). Scrotal ultrasound showed no abnormalities in the bilateral testis or epididymis. Chest CT showed no significant abnormalities in bilateral lungs.

**Figure 2:**
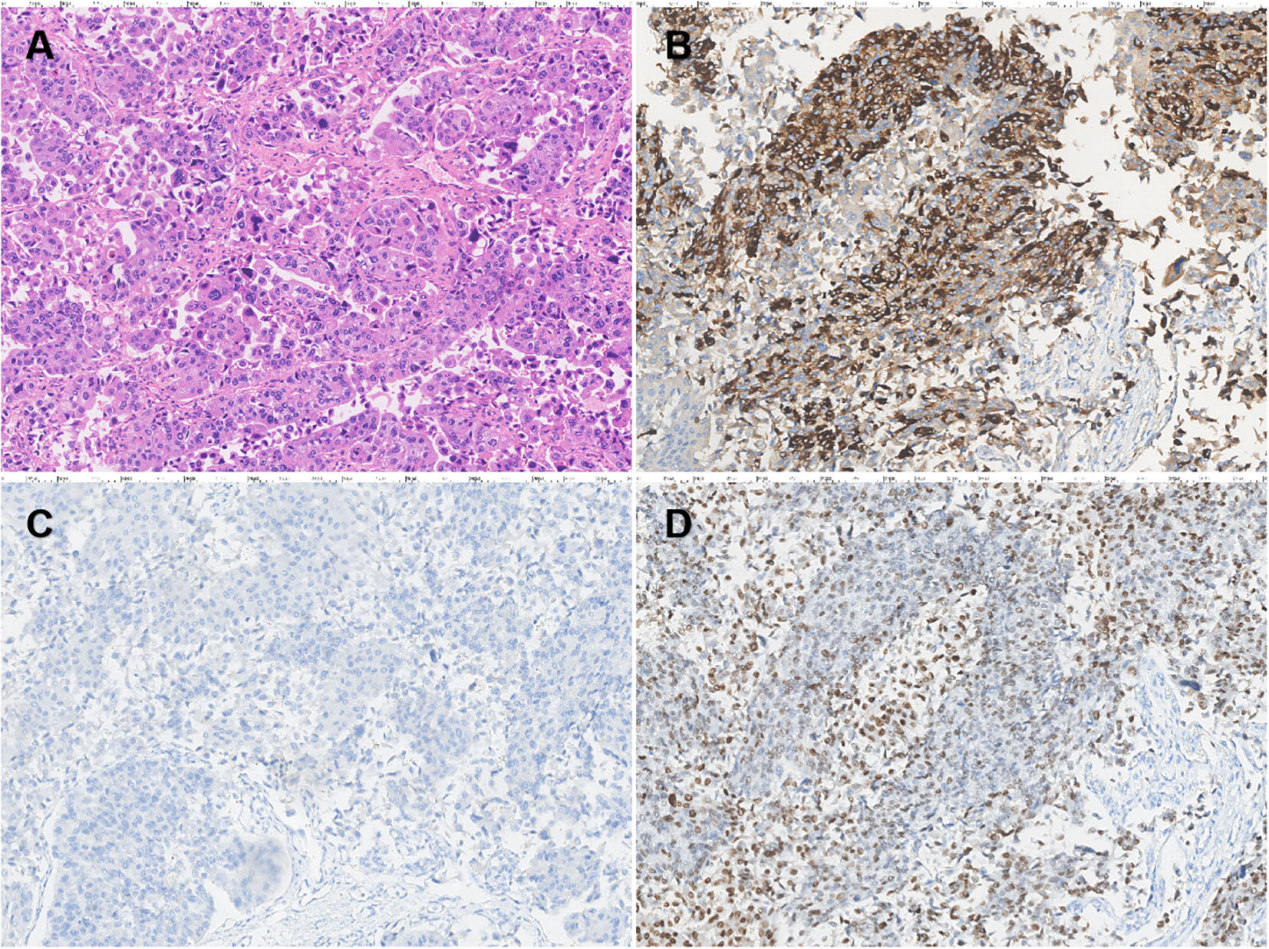
Pathological analysis of bladder carcinoma (×200). H&E staining of tissue sections revealed a high-grade invasive urothelial carcinoma of the bladder **(A)**. Immunohistochemistry revealed strongly positive AFP expression in urothelial carcinoma of the bladder **(B)**. Hepatocyte staining was negative **(C)** and GATA3 staining was strongly positive **(D)**.

Cystoscopy under epidural anesthesia revealed multiple cauliflower-like tumors on the bladder walls, some with calcifications, and involvement of the bladder neck and prostatic urethra. A diagnostic transurethral resection of the bladder tumor (TURBT) was performed, and pathology confirmed high-grade invasive urothelial carcinoma. A multidisciplinary team (MDT) discussion was organized, and experts postulated that the elevated AFP may have been caused by the bladder tumor. Although the tumor was at a later stage, the patient had no metastasis and the tumor was resectable, recommending surgery as the first choice. The patient then underwent robotic-assisted laparoscopic radical cystectomy with total urethrectomy and ileal conduit urinary diversion. The surgery was successful, and the postoperative pathology showed high-grade muscle invasive bladder urothelial carcinoma (**Figure 2A**), with the maximum tumor diameter measuring approximately 7.5cm, invading the full thickness of the bladder wall, and showing vascular and perineural invasion. There was no evidence of cancer involvement in the prostate, urethra, or resection margin of the bilateral vas deferens, except for vascular invasion in the left seminal vesicle. No cancer involvement was found in the bilateral ureteral stump, and there was no metastasis in the left (0/13) and right (0/28) pelvic lymph nodes. The tumor stage was T4aN0M0, stage IIIA. Immunohistochemistry revealed that the patient was MLH1 (+), PMS2 (+), MSH2 (+), and MSH6 (+) positive, indicating proficiency in mismatch repair (pMMR), and also that the patient was HER-2 (2+) and Ki67 (about 10%+) positive. AFP staining was strongly positive (**Figure 2B**), indicating that the tumor produced AFP. Hepatocyte staining was negative (**Figure 2C**), excluding hepatoid differentiation, and GATA3 staining was strongly positive (**Figure 2D**), supporting urothelial origin.

**Figure 3:**
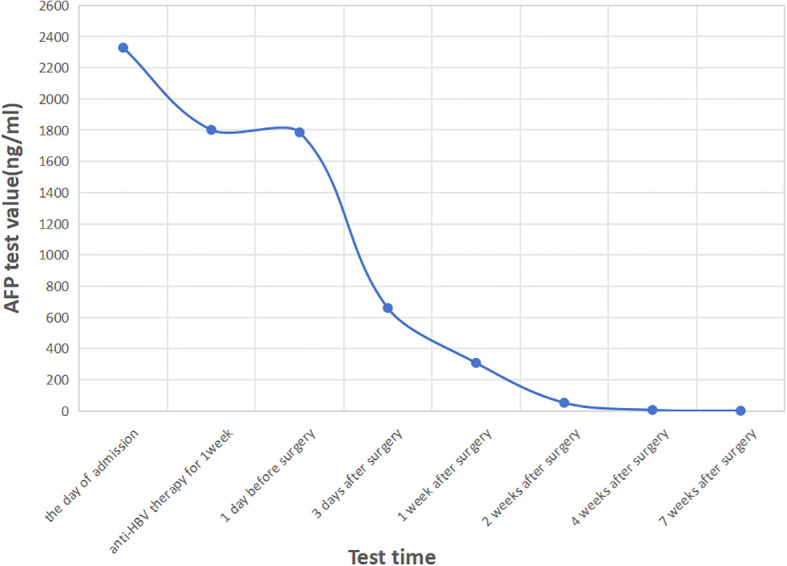
The curve of AFP levels over time. After surgery, the serum AFP levels dropped sharply and decreased to normal 4 weeks after the operation.

Serum AFP levels dropped significantly postoperatively without further anti-HBV treatment: 660 ng/ml on day 3, 308 ng/ml at week 1, 52.5 ng/ml at week 2, 5.99 ng/ml at week 4, and <0.91 ng/ml at week 7 (**Figure 3**). The bilateral ureteral stents were removed 3 months postoperatively. Liver and renal function remained normal throughout the hospitalization. We have repeatedly suggested the patient undergo chemotherapy with GC regimen, targeted therapy with ADC drugs, or immunotherapy with PD-1 inhibitors after the surgery, but due to concerns about the side effects of the medication and financial constraints, the patient did not receive any adjuvant therapy. At the 1-year follow-up, the patient was in good condition, with no evidence of tumor recurrence or metastasis on chest and abdominal CT scans.

## Discussion

AFP is derived from embryonic stem cells and synthesized by the fetal liver, yolk sac, and gastrointestinal tract before birth. AFP expression disappears approximately 2 weeks after birth. Increased serum AFP levels later are primarily associated with HCC, especially when levels are above 200 ng/ml (4). However, it is not an accurate single diagnostic criterion for liver cancer. Serum AFP elevation may also occur in viral hepatitis, liver cirrhosis, and other AFP-producing tumors including non-germ cell tumors (5), gastric cancer, ovary cancer, and lung cancer (1–3). The absence of AT motif-binding factor 1 may play a role in AFP-producing gastric cancer, as reported by Kataoka et al. (6).

In our case, the patient’s serum AFP was significantly high. Although he was positive for HBsAg, HBeAb, and HBcAb, his HBV-DNA quantitative result was not very high and his liver function was normal. Furthermore, after anti-HBV treatment for 1 week before surgery, his serum AFP remained significantly elevated at 1,802 ng/ml. We excluded the common diseases that would lead to AFP elevation. CEA, CA125, CA153, and CA199 were all in normal limits. There were no relevant abnormalities on ultrasound, CT, and MRI examinations, except for bladder cancer. Considering the priority to treat the bladder tumor, surgery was performed first. After radical cystectomy, his serum AFP levels dropped sharply without further anti-HBV treatment and returned to normal in 4 weeks. Immunohistochemical staining of the tumor tissue was strongly positive for AFP while negative for hepatocytes. As a result, we presume that the elevated serum AFP was produced by the bladder cancer tissue.

AFP-producing urothelial carcinoma is rarely reported in the literature. AFP-producing hepatoid adenocarcinoma of the bladder has been reported in several cases (7–9). These hepatoid adenocarcinoma cases displayed hepatoid differentiation, such as the formation of cords of polygonal cells, bile canaliculi formation, or evidence of bile production, and positive AFP staining (10). The histogenesis of hepatoid adenocarcinoma may originate from a multi-potent cell or the differentiation of tumor cells in an endodermal direction. According to the published literature, only one case of urothelial carcinoma of the renal pelvis and three cases of the urinary bladder have been reported (11–14). This is the fourth case of urothelial carcinoma of the bladder with AFP elevation. In this case, histological examination showed high-grade invasive urothelial carcinoma without obvious hepatoid differentiation. The GATA3 positivity indicated the urothelial origin of the tumor. The serum AFP levels fell rapidly after the operation, indicating an AFP-producing urothelial carcinoma of the urinary bladder. Pectasides et al. (15) retrospectively evaluated the serum tumor markers for bladder urothelial carcinomas and concluded that AFP is not an effective marker for the diagnosis, response prediction, and monitoring of bladder urothelial carcinoma. Serum AFP testing is not routinely performed in patients with bladder cancer. Thus, the rare case reports may be due to this fact.

It is reported that bladder urothelial carcinomas with squamous or glandular differentiation are more aggressive and have a poorer prognosis than general urothelial carcinomas (16). Tumors with hepatoid features, regardless of AFP production, also have a poor prognosis (10). It is also reported that an AFP-producing bladder transitional cell carcinoma (TCC) with lung and bone metastases behaved aggressively and had a poor prognosis (13). Up to now, only one case of AFP-producing bladder TCC has been successfully controlled by radiotherapy alone, as reported by Shiga et al. (11), and another was successfully controlled by radical cystectomy, as reported by Jianan Ye et al. (14). In our case, the tumor stage was T4aN0M0, stage IIIA, but the preoperative examination did not reveal tumor metastasis and the tumor was resectable, therefore radical cystectomy was recommended as the first choice. Furthermore, the patient and his family also prioritized and requested surgical treatment. The operation was successful and the postoperative pathology results indicated negative surgical margins and no lymph node metastasis. We recommended that the patient undergo chemotherapy with the GC regimen of cisplatin combined with gemcitabine after surgery. However, the patient and his family believe that the side effects of chemotherapy are too severe, especially considering the patient’s weakened condition following such a major surgery, and therefore they are reluctant to proceed with chemotherapy. Given that the patient’s immunohistochemical results indicated HER2 2+, we considered the possibility of a targeted immunotherapy combination treatment with an ADC drug (viditilimab) and a PD-1 inhibitor (such as toripalimab). However, due to health insurance policies, this combination cannot be reimbursed as a first-line treatment, and the patient is unable to afford the out-of-pocket expenses. Therefore, no adjuvant therapy has been pursued following the surgery. Currently, the patient has been followed for a year, and there was no tumor recurrence or metastasis. Because the current follow-up time was only 1 year, we could not determine the prognosis and survival time, however, we will continue to closely follow this patient.

## Conclusion

AFP-producing urothelial carcinoma of the bladder is an extremely rare entity. The nature of this carcinoma remains unknown. Whether an AFP elevation affects the response and prognosis of bladder urothelial carcinoma is still unclear and requires further investigation.

## Data Availability

The original contributions presented in the study are included in the article/supplementary material. Further inquiries can be directed to the corresponding author.
